# Dynamic chromatin architectures provide insights into the genetics of cattle myogenesis

**DOI:** 10.1186/s40104-023-00855-y

**Published:** 2023-04-14

**Authors:** Jie Cheng, Xiukai Cao, Xiaogang Wang, Jian Wang, Binglin Yue, Wei Sun, Yongzhen Huang, Xianyong Lan, Gang Ren, Chuzhao Lei, Hong Chen

**Affiliations:** 1 College of Animal Science and Technology, Northwest A&F University, No.22 Xinong Road, Yangling district, Yangling, Shaanxi province 712100 China; 2 Joint International Research Laboratory of Agriculture and Agri-Product Safety of Ministry of Education of China, Yangzhou University, Yangzhou, 225009 China; 3 Key Laboratory of Qinghai-Tibetan Plateau Animal Genetic Resource Reservation and Utilization, Southwest Minzu University, Chengdu, 610225 China; 4 College of Animal Science and Technology, Yangzhou University, Yangzhou, 225009 China; 5 College of Animal Science, Xinjiang Agricultural University, Urumqi, 830052 China

**Keywords:** Cattle, *Cis*-regulatory elements, Muscle, Selection sweep, 3D genome

## Abstract

**Background:**

Sharply increased beef consumption is propelling the genetic improvement projects of beef cattle in China. Three-dimensional genome structure is confirmed to be an important layer of transcription regulation. Although genome-wide interaction data of several livestock species have already been produced, the genome structure states and its regulatory rules in cattle muscle are still limited.

**Results:**

Here we present the first 3D genome data in *Longissimus dorsi* muscle of fetal and adult cattle (*Bos taurus*). We showed that compartments, topologically associating domains (TADs), and loop undergo re-organization and the structure dynamics were consistent with transcriptomic divergence during muscle development. Furthermore, we annotated *cis*-regulatory elements in cattle genome during myogenesis and demonstrated the enrichments of promoter and enhancer in selection sweeps. We further validated the regulatory function of one *HMGA2* intronic enhancer near a strong sweep region on primary bovine myoblast proliferation.

**Conclusions:**

Our data provide key insights of the regulatory function of high order chromatin structure and cattle myogenic biology, which will benefit the progress of genetic improvement of beef cattle.

**Supplementary Information:**

The online version contains supplementary material available at 10.1186/s40104-023-00855-y.

## Background

As an expanding beef market, China has registered a 19-fold growth in per capita beef consumption from 0.31 kg in 1978 to 6.28 kg in 2019 (original data from FAO and further analyzed by Our World in Data, https://ourworldindata.org/grapher/per-capita-meat-type). This sharp increase with the resulting beef deficit has become principle motives behind the breeding up projects of beef cattle in China. To this day, several representative cattle breeds have been genetically improved for the land [1]. However, better genetic quality may be further achieved through exploring the molecular basis of skeletal myogenesis [2–4].

Three-dimensional genome organization is now confirmed as an additional layer in gene regulation [5, 6]. Chromosomes are not randomly distributed in the nuclear space but instead occupy discrete volumes called chromosome territories as revealed by fluorescence in situ hybridization [7, 8]. Recently, by measuring the frequency of physical interactions between genomic loci, various ‘chromosome conformation capture technologies’ confirmed the existence of chromosome territories at a resolution of several megabase and identified extra hierarchical structure units of chromosomes at higher resolutions [9, 10]. Compartments have transcriptional active A and inactive B forms and the two forms alternate along the length of genome, which broadly correspond to euchromatin and heterochromatin, respectively [11, 12]. Topologically associating domains (TADs) are formed by loop extrusion and defined as highly self-interaction regions [13]. The TAD boundaries restrict the interactions within the same TAD, while insulating interactions within different domains [14, 15]. TAD disruption can lead to enhancer adoption and misregulation of essential genes [16–18]. These genome structures commonly exist in higher eukaryotes and the dynamics of conformational genome has been functionally associated with development and cell differentiation of human and model organisms [19–22], however, very limited data are available for livestock genomes [4].

It is confirmed that the *cis*-regulatory elements may be indispensable drivers of phenotypic variation of domesticated animals, given the facts that most genome-wide association study (GWAS) hits lie outside protein-coding regions [2, 23]. Chromatin loops, the most fine-scaled conformation of 3D genome, serve as bridges between transcriptional regulation and phenotypic variation by bringing regulatory elements and their target genes into close spatial proximity [2, 24]. To date, several studies have deciphered novel enhancers of muscle genes for human [25], mouse [26–29], and pig [21], by calling loops from high-resolution chromatin interaction maps. Moreover, the Functional Annotation of Animal Genomes consortium is working to create reference functional maps of farmed animals by profiling the landscape of transcription, chromatin accessibility and genome conformation [3]. However, the spatial organization of cattle muscle genome involving regulatory elements and its impact on gene expression are still lacking [30], representing a critical knowledge gap from the genetics to phenotype of cattle muscle development.

In this study, we employed RNA sequencing (RNA-seq), assay for transposase accessible chromatin with high-throughput sequencing (ATAC-seq), and high-throughput chromosome conformation capture (Hi-C) methodologies to characterize the dynamics and functions of the 3D genome structure during muscle development. We further evaluated the enrichment of selection sweeps for regulatory sequences and revealed the regulatory function of one *HMGA2* intronic enhancer near the strongest selection signal on primary bovine myoblast proliferation. Our data provide some novel insights into the molecular basis of cattle myogenesis.

## Methods

### Skeletal muscle samples

*Longissimus dorsi* muscle from twelve fetuses and five adult cows of Qinchuan cattle (QC) were collected for high-throughput sequencing or primary myoblast isolation. The fetal samples and adult samples were obtained from slaughterhouses in Shannxi province and Gansu province (China), respectively. All the slaughtered cows are commercial individuals and genetically unrelated. The experimental protocols were approved by the Institutional Animal Care and Use Committee of Northwest A&F University (NWAFAC1020).

### Hi-C library construction and sequencing

We generated Hi-C data from two adult samples (about 2 years old) and two female fetal samples (about 2 months old) with similar body size between the replicates. Hi-C libraries were prepared following the previously published protocol with minor modifications compatible with frozen tissue [11]. Briefly, one gram of tissue was grinded into a fine powder and digested with collagenase I (Gibco BRL, Grand Island, NY, USA) at 37 °C for 1 h to guarantee the production of more than 2 × 10^6^ valid cells. Cross-linking was performed with formaldehyde (a final concentration of 2%) for 15 min at room temperature (RT) and quenched with glycine (a final concentration of 0.2 mol/L) for 5 min at RT, followed by 15 min on ice. The cell suspension was then centrifuged at 1500 r/min for 10 min at RT to get cell pellet. After incubated with 550 μL lysis buffer (500 μL 10 mmol/L Tris-HCl, 10 mmol/L NaCl, 0.2% Igepal CA-630 and 50 μL protease inhibitors) on ice for 15 min, the suspension was spun down for 5 min at 5000 r/min at RT. Nuclei were permeabilized with 38 μL 1% SDS for 10 min at 65 °C and then quenched with 44 μL 10% Triton X-100. Chromatin was subsequently digested overnight (o/n) at 37 °C by adding 400 Units *Mbo*I (NEB, Knowl Piece, Hitchin, UK) followed by labeling of the DNA fragment ends with biotin (1.5 μL 10 mmol/L dATP, 1.5 μL 10 mmol/L dGTP, 1.5 μL 10 mmol/L dTTP, 37.5 μL 0.4 mmol/L biotin-14-dCTP and 10 μL 5 U/μL Klenow) through incubation at 37 °C for 45 min. Enzymes were inactivated by adding 86 μL 10% SDS and incubating tube at 65 °C for 30 min. After adding 7.61 mL ligation mix (745 μL 10% Triton X-100, 745 μL 10× ligation buffer, 80 μL 10 mg/mL BSA, 80 μL 100 mmol/L ATP and 5.96 mL water) and 50 μL 1 U/μL T4 DNA ligase, the reaction was incubated at 16 °C o/n. To reverse crosslinks and to degrade protein, 50 μL 10 mg/mL proteinase K was added and then incubated overnight at 65 °C. This was followed by DNA purification with phenol-chloroform extraction and fragmentation to 300–500 bp with the Covaris S220 ultrasonicator. The DNA was then pulled down with 75 μL Dynabeads M-280 Streptavidin (Thermo Fisher Scientific, Waltham, MA, USA). Libraries were constructed following Illumina protocols and sequenced on an Illumina HiSeq 2500 PE150 platform.

### ATAC-seq library construction and sequencing

An improved ATAC-seq protocol reducing background and allowing interrogation of frozen tissues was used [31]. Briefly, 20 mg frozen muscle were thawed for 5 min on ice in cold Homogenization Buffer followed by Dounce homogenization. Pre-clear larger chunks by pelleting at 100 × *g* for 1 min in a pre-chilled centrifuge. Then density gradient centrifugation with 25%, 29%, 35% Iodixanol solution were carried out for 20 min at 3000 × *g* to isolate nuclei. The nuclei were transferred to a tube containing 1 mL of ATAC-seq RSB (10 μL 1 mol/L Tris-HCl pH 7.4, 2 μL 5 mol/L NaCl, 3 μL 1 mol/L MgCl_2_, 985 μL H_2_O) with 0.1% Tween-20 and then spun for 10 min at 500 × *g* at 4 °C. After removing the supernatant, transposition reaction was performed in ATAC-seq reaction mix (25 μL 2× TD buffer, 2.5 μL transposase, 16.5 μL PBS, 0.5 μL 1% digitonin, 0.5 μL 10% Tween-20, 5 μL H_2_O) at 37 °C for 1 h. DNA was purified with a MinElute PCR Purification Kit (Qiagen). The transposed DNA fragments were amplified for 5 cycles (NEB, Knowl Piece, Hitchin, UK) and the additional cycles were determined by qPCR. The concentrations of purified libraries were > 2 nmol/L, which was quantified using the KAPA Library Quantification Kit (Roche Sequencing Solutions, Pleasanton, CA, USA). The libraries were sequenced on an Illumina HiSeq 2500 PE150 platform.

### RNA-seq library construction and sequencing

Total RNA was extracted using TRIzol reagent (Thermo Fisher Scientific, Waltham, MA, USA). RNA degradation and contamination was monitored on 1% agarose gels. RNA purity was checked using spectrophotometer. RNA integrity was assessed using the RNA Nano 6000 Assay Kit of the Bioanalyzer 2100 system (Agilent Technologies, Santa Clara, CA, USA). A total amount of 1 μg high-quality RNA was used as input material for the library preparation. Sequencing libraries were generated using NEBNext UltraTM RNA Library Prep Kit for Illumina (NEB, Knowl Piece, Hitchin, UK) following manufacturer’s recommendations and index codes were added to attribute sequences. The libraries were sequenced on an Illumina HiSeq 2500 PE150 platform.

### Hi-C data analysis

#### Mapping and matrix generation

Configuration file was first prepared for HiC-Pro [32] pipeline v2.9.0. BOWTIE2_IDX_PATH was the bowtie2 v2.4.3 [33] indexes of reference genome (ARS-UCD1.2). GENOME_FRAGMENT was the bed file with restriction fragments generated from digest_genome.py with the parameter “-r ^GATC”. LIGATION_SITE was set as GATCGATC. The paired-end Hi-C reads from different libraries of the same sample were put in the same folder and mapped using HiC-Pro [32] pipeline v2.9.0 with the parameter “-s mapping”. The obtained bam file was then used to filter invalid pairs with the parameter “-s proc_hic”, including singletons and multi-hits, dangling end, dumped and self- circles pairs, PCR duplication. The generated allValidPairs file was applied to build raw inter-/intra-chromosomal contact map with the parameter “-s build_contact_maps”, followed by iterative correction and eigenvector decomposition (ICE) normalization on matrix file with the parameter “-s ice_norm”. After confirming a very high correlation between the ICE normalized matrices at 200 kb resolution, we merged the valid pairs of the corresponding replicates into a single file with the parameter “-s merge_persample”. The merged file was used as input data to rebuild normalized matrices at resolutions of 10 kb, 40 kb and 1 Mb using HiC-Pro [32] pipeline v2.9.0 and generate hic file at resolutions of 5 kb, 10 kb, 25 kb, 50 kb, 100 kb, 250 kb, 500 kb, 1 Mb and 2.5 Mb using hicpro2juicebox.sh for juicer tools v1.9.8 [34].

#### A/B compartment forms

The eigenvector is the first principal component of the Pearson’s matrix and can be used to delineate compartments in Hi-C data. The juicer tools v1.9.8 [34] was used to call compartment with the parameter “BP 1000000” for the hic files.

#### TAD

Most upstream portion of a TAD is highly biased towards interacting downstream, and the downstream portion of a TAD is highly biased towards interacting upstream. Here, we exploited the directionality index (DI) by identifying such biases in interaction frequency in the genome as previously described [35, 36]. We set 40 kb bin to the upstream/downstream 2 Mb. After calculating the DI of ICE matrix, a hidden Markov model was used to identify biased states (“Upstream Bias”, “Downstream Bias” or “No Bias”) with the parameters (thresholds of median probabilities and minimal size for probability correction) as their default values (0.99 and 2, respectively). Regions between TADs within 400 kb were identified as TAD boundaries.

#### Loop

Chromatin loops show up as dots/points on a Hi-C contact map. We used HICCUPS [34] to identify genome-wide loops using hic files with the parameter “-r 5000, 10,000”. Stage-specific loops were analyzed by HiCCUPSDiff [34] using the identified loops and hic files with default parameter. Aggregate peak analysis [34] was performed to measure the aggregate enrichment of different loops in a contact matrix [37] using the different loops and hic files with the parameter “-r 5000”. We used Fit-Hi-C v1.1.3 [38, 39] to find significant interactions of *cis*-regulatory elements with *HMGA2* (false discovery rate, FDR *q-*value < 0.05). Chromosome 5 (Chr5) interactions were extracted from the genome matrix (10 kb) as input file for HiCPro2FitHiC.py with the parameter “-r 10,000” followed by running HiCKRy.py with default parameter. Finally, significant interactions were called with the parameter “-r 10000 -p 2”.

### ATAC-seq data analysis

#### Mapping

Adapters and low quality (phred quality < 10) bases were removed from raw sequencing reads with Trimmomatic v0.38 [40] and the trimmed reads were aligned to reference genome (ARS-UCD1.2) using bowtie2 v2.4.3 [33] with the parameter “-X 2000”. High quality paired alignments (mapping quality ≥ 30) were extracted with samtools v1.9 [41] after filtering unmapped reads, mate unmapped reads, not primary alignments, reads failing platform. To generate valid pairs for peak calling, PCR duplications and organelle contamination were further removed by Picard v1.126 (https://broadinstitute.github.io/picard) and bedtools v2.26.0 [42] with default parameters. The bam files were converted to bigwig files to be visualized in IGV [43].

#### Insert size and transcription start site (TSS) enrichment

The insert size distribution has clear periodicity of approximately 200 bp, suggesting many fragments are protected by integer multiples of nucleosomes, while reads from nucleosome-free regions were enriched at 40–60 bp [44]. To evaluate the chromatin integrity for subsequent analysis, we first detected insert size distribution. Transcribed promoter-flanking regions are usually open and enriched with ATAC-seq reads. Therefore, we next calculated the TSS enrichment scores using the ENCODE script (https://github.com/ENCODE-DCC/atac-seq-pipeline/blob/master/src/encode_task_tss_enrich.py). Briefly, read counts around TSS (± 3 kb) were summed per bin (400 bp) after shifting 75 bp toward 5 primes of each read and then extending to 150 bp uniformly and then the average read counts of all transcripts in each bin were calculated. The number of the bin which overlapped with TSS was taken as TSS enrichment score.

#### Peak calling and data reproducibility

MACS2 v2.1.0 [45] was used for peak calling with following parameter “--nomodel --shift -75 --extsize 150” after converting alignments from bam to bed format according to the guidelines of the ATAC-seq pipeline from the ENCODE project (https://github.com/kundajelab/atac_dnase_pipelines). The irreproducible discovery rate method was used to assess the consistency of replicate peak sets [46]. The peaks of two replicates were merged using idr v2.0.2 (https://github.com/nboley/idr). Next, the two common peak sets were combined to form a union peak set according to the criteria that individual peaks were merged if overlap ≤ 10 bp using bedtools v2.26.0 [42] with parameter of “bedtools multiinter” followed by “bedtools merge -d 10”. The number of reads of each sample at the union intervals were re-called with the parameter of “bedtools multicov -bams” to generate count matrix. For each union peak, its enrichment value is defined as the ATAC-seq signal intensity (normalized read count per base) subtracted from the background noise (normalized read count per base). The count matrix was used as input file of DESeq2 v1.32.0 [47] to call differentially accessible regions (DARs, *P*-value < 0.05). Motif enrichment analysis was performed with the MEME Suite (https://meme-suite.org/meme/).

#### Sample correlation

 Enrichment value listed in the union peak set were used to analyze the principal components analysis (PCA) and Pearson correlation coefficient of the four samples. PC1 and PC2 accounted for 65.2% variance and 25.6% variance, respectively, indicated by FactoMineR v2.4 [48].

### RNA-seq data analysis

#### Mapping

Clean reads were obtained by removing low quality reads from raw data, including reads with adapter, undetermined bases, and reads with more than 50% low quality bases (phred quality < 20) using Trimmomatic v0.38 [40]. We gained 97.8%–98.7% clean reads from raw data for the twelve samples. Next, the obtained clean reads were subsequently mapped to reference genome (ARS-UCD1.2) using Hisat2 v2.0.5 [49] with default parameters. Gene model annotation file was retrieved from NCBI (https://www.ncbi.nlm.nih.gov/genome/?term=Bovine) as well. Hisat2 can generate a data set of splice junctions based on the gene model annotation file and thus has a better mapping result than other non-splice mapping tools [49]. After filtering unmapped reads and multi-mapped reads, the clean data produced about 93% unique reads for each sample which were used for downstream analyses. The sam files were converted to bam files with samtools v1.9 [41] to be visualized in IGV [43].

#### Prediction of novel transcripts

The unique reads of each sample were assembled by StringTie v1.3.3b [50]. StringTie uses a de novo assembly step to assemble and quantitate full length transcripts representing multiple splice variants for each gene locus [50]. A total of 715 novel transcripts were identified with the maximum fragments per kilobase of transcript per million reads mapped (FPKM) of 560. Functional annotation of these novel transcripts was performed as previously described [51].

#### Quantification of gene expression

Before quantifying gene expression, low quality alignment (< 10) reads and unpaired reads were removed. Next, featureCounts v1.5.0-p3 [52] was used to count the read number mapped to each gene and FPKM was calculated. The identification of differential expression gene (DEG) was performed using DESeq2 v1.32.0 [47] with FDR *q*-value < 0.05.

### Characterization of loops

Loop anchors identified by HICCUPS [34] at 5 kb and 10 kb resolutions (merged files) were intersected with ATAC-seq peaks using bedtools v2.26.0 [42]. We carried out a sequential classification scheme to sort the ATAC-seq peaks into promoter (P), enhancer (E), and other (O) based ATAC-seq union peak set. The ±3 kb windows of the TSSs of all expressing genes (mean FPKM of the twelve samples > 0 as determined from RNA-seq data) were used to intersect with ATAC-seq union peaks and the overlapped peak set was defined as promoter. Next, PSYCHIC [53] was applied to identify enhancer candidates. Notably, PSYCHIC is designed to predict promoter-enhancer interaction within TAD and thus spatial contacts at the TAD boundaries will escape annotation [17, 53]. We extracted matrix for each chromosome at 25 kb resolution from hic file using juicer tools v1.9.8 [34] with the parameter “dump observed KR BP 25000”, followed by transforming the obtained sparse upper triangular matrix to a full contact matrix as input file for PSYCHIC using HiCcompare v 1.14.0 [54]. Promoter-enhancer interactions were then predicted using htad-chain.py with the parameter “res: 25000 win: 2000000” in configuration file. The predicted enhancers (FDR *q*-value < 0.05) were used to intersect with ATAC-seq union peaks (promoter peaks excluded) and the overlapped peak set was defined as enhancer. After filtering promoters and enhancers, the remaining ATAC-seq union peak set was defined as other regulatory elements. Loop anchors without any peaks were defined as none regulatory elements (N).

### Genome scanning for selection sweeps

A total of 86 cattle of 7 breeds with low beef production and 3 internationally renowned beef breeds were collected. The low production group contained Bashan (*n* = 5), Dabieshan (*n* = 2), Jiaxian (*n* = 1), Lingnan (*n* = 8), Nanyang (*n* = 2), Weining (*n* = 5), and Zaobei (*n* = 3). Their brief introductions can be retrieved from Catalogue of National Livestock and Poultry Genetic Resources (https://zypc.nahs.org.cn/pzml/). The high production group included Angus (*n* = 25), Charolais (*n* = 14), and Hereford (*n* = 21).

To call single nucleotide polymorphisms (SNPs), we mapped clean reads to reference genome (Btau_5.0.1) using BWA-MEM v0.7.13-r1126 [55] with default parameters, followed by the removal of duplicate reads with Picard v1.126 (https://broadinstitute.github.io/picard). GATK v3.6–0-g89b7209 [56] was applied to detect SNPs according to the previously described criteria [57]. SNPs with maximum missing rate < 0.3 and minor allele frequency > 0.01 were extracted by VCFtools v0.1.16 [58] and used for subsequent analyses. PCA was carried out using eigensoft with default parameters (https://github.com/chrchang/eigensoft). Population differentiation was measured as the fixation index (*F*_ST_) values using VCFtools v0.1.16 [58] with the parameter “--fst-window-size 10000 --fst-window-step 5000”. Window-size *F*_ST_ values were then transformed to *ZF*_*ST*_ = (*F*_*ST*_ − μF_ST_)/σ*F*_*ST*_. The regions were remapped to reference genome ARS-UCD1.2 by NCBI Genome Remapping Service (https://www.ncbi.nlm.nih.gov/genome/tools/remap). Metascape [59] and GREAT v4.0.4 [60] were used for functional annotation of coding genes and regulatory elements within selection sweeps, respectively. We also analyzed the enrichment of 2045 sweep regions (*ZF*_*ST*_ > 3, top ~ 0.4%) for regulatory elements using LOLA v1.22.0 [61]. The input files were prepared as followings: the four kinds of regulatory elements were used as “query set”; the 2045 sweep regions were used as “reference set”; 10 kb genome-wide bins (remapped to ARS-UCD1.2) were used as “Universe Set”.

### Cell culture and treatment

Primary bovine myoblasts (PBMs) were isolated from *Longissimus dorsi* of fetal cattle by collagenase II as previously described [62]. PBMs were cultured with growth medium made up of DMEM (Gibco BRL, Grand Island, NY, USA) supplemented with 20% FBS (Thermo Fisher Scientific, Waltham, MA, USA) and 1% double antibiotics at 37 °C in 5% CO_2_. Recombinant plasmids were transfected into PBMs using TurboFect Transfection Reagent (Thermo Fisher Scientific, Waltham, MA, USA) when the cells grew to 50% ~ 60% confluence, followed by 24 h incubation.

### DNA and RNA preparation

With the standard phenol-chloroform method, we extracted genomic DNA from 1 mL 2% heparin-treated QC whole blood samples. Total RNA was extracted from PBMs using TRIzol reagent. Next, 1 μg RNA was reversely transcribed for cDNA synthesis using PrimeScript RT reagent Kit with gDNA Eraser (TaKaRa, Dalian, Liaoning, China) following the instruction.

### Dual-luciferase reporter assay

The QC genomic DNA was used for PCR amplification of enhancer candidate (RE). Negative control region without any ATAC-seq peaks was selected to confirm the enhancer activity of RE. The amplified products were purified with SanPrep Column PCR Product Purification Kit (Sangon Biotech, Shanghai, China). Next, the purified products and pGL3-Promoter plasmid (firefly luciferase) were digested by *BamH* I and *Sal* I (TaKaRa, Dalian, Liaoning, China) and separated on a 1% agarose gel, followed by purification with SanPrep Column DNA Gel Extraction Kit (Sangon Biotech, Shanghai, China). The products were ligated with T4 DNA ligase (TaKaRa, Dalian, Liaoning, China) at 16 °C o/n. The recombinant plasmids were confirmed by Sanger sequencing (Sangon Biotech, Shanghai, China) and co-transfected with pRL-TK plasmid (*Renilla* luciferase). After 24 h incubation, luciferase activities were measured with Dual-Luciferase Reporter Assay Systems (Promega, Madison, WI, USA). Briefly, the cells were harvested using 50 μL of 1× passive lysis buffer and lysed in 96-well plates for 40 min. Next, 20 μL of Luciferase Assay Reagent II was added to quantify firefly luciferase activity, followed by the addition of 20 μL of Stop & Glo Reagent. Blank pGL3-Basic plasmid, blank pGL3-Promoter plasmid, and pGL3-Control plasmid were used as blank control, negative control, and positive control, respectively. A Microplate Reader was used to qualify the luciferase activities of five replicates of each group and the firefly luciferase activity was normalized against *Renilla* luciferase activity.

### CRISPRi assay

CRISPR interference (CRISPRi) mediated enhancer repression was performed with pX330a Cas9-KRAB vector (Addgene #92361, www.addgene.org) [63]. CRISPOR (http://crispor.tefor.net/) was used to design short guide RNAs (sgRNAs). Twenty microliters of primer pair (100 μmol/L) and 80 μL H_2_O were heated at 95 °C for 5 min and cooled down to RT to form dimer. The pX330a Cas9-KRAB vector was digested with *Bbs* I (TaKaRa, Dalian, Liaoning, China) and recovered with gel extraction. Next, the products were ligated with the dimer using T4 DNA ligase (TaKaRa, Dalian, Liaoning, China) at 16 °C o/n. The confirmed recombinant vectors were transfected into PBMs at 50% ~ 60% confluence which grew in 6-well plates and cells were collected after 24 h incubation.

### RT-qPCR

The diluted cDNA (10 ng/μL) was used to perform qPCR amplification using the SYBR Premix Ex Taq II Kit (TaKaRa, Dalian, Liaoning, China) with nine replicates. Relative expression level was calculated by 2^-△△CT^ method and data were normalized to *GAPDH* mRNAs.

### EdU assay

This assay relies on incorporation of 5-ethynyl-2′-deoxyuridine (EdU) into de novo DNA synthesized during the S-phase of the cell cycle. EdU assay was carried out with Cell-Light EdU Apollo 567 In Vitro Imaging Kit (RiboBio, Guangzhou, Guangdong, China) according to the manufacturer’s instructions. Briefly, PBMs were seed onto 96-well plates and cultured to 80% confluence in 100 μL growth medium. One hundred milliliters of EdU (20 μmol/L final concentration) was added to each well followed by incubation at 37 °C for 2 h. Cells were fixed using 50 μL 4% formaldehyde for 20 min at RT. Next, we removed the supernatant and added 50 μL 2 mg/mL glycine, followed by incubation for 5 min at RT. Cells were permeabilized with 100 μL 0.5% Triton X-100 and incubated for 20 min at RT. Add 100 μL 1× Apollo staining solution and incubated for 30 min at RT without light. After washed with PBS, the cells were incubated with 100 μL 1× Hoechst33342 for 30 min at RT. Finally, cell nuclei were detected by fluorescence microscopy.

### Flow cytometry

Cell cycle staining Kit (Multisciences, Hangzhou, Zhejiang, China) was used for this experiment following the manufacturer’s instructions. Briefly, PBMs were seed onto 6-well plates with 2 mL growth medium and collected at 80% confluence. The pellet was resuspended by 1 mL cold 70% ethanol diluted with PBS, followed by the incubation at −20 °C o/n. Next, cells were centrifuged at 1000 r/min for 5 min, followed by the addition of 1 mL DNA Staining solution and 10 μL Permeabilization solution. Samples were incubated at 4 °C for 30 min away from light. Cell cycles were detected by a flow cytometry with three replicates of each group.

## Results

### Comprehensive maps of chromatin contacts in fetal and adult cattle muscle

**Fig. 1:**
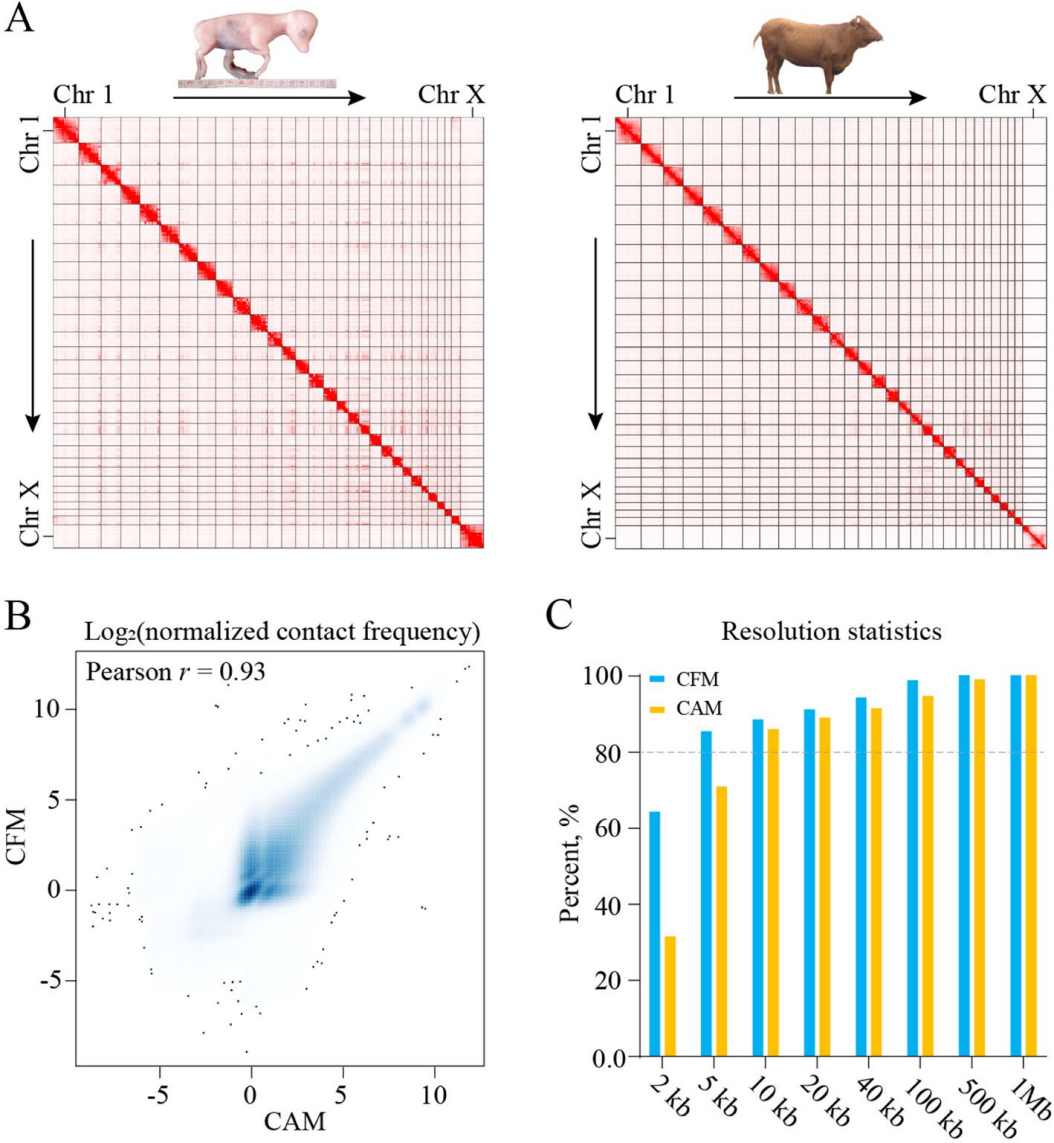
3D genome comparisons of fetal and adult cattle *Longissimus dorsi* muscle. **A** Hi-C contact heatmaps (left: CFM, right: CAM) visualized by JuiceBox. Each dot on heatmaps represented the observed number of read pairs. **B** Pearson’s correlation of ICE matrices at 40 kb. **C** Analysis of resolution capability by determining the smallest bin size where 80% of bins have at least 1000 valid contact pairs. CFM, fetal cattle muscle; CAM, adult cattle muscle

To construct and compare chromatin structures between fetal and adult stages, we determined genome-wide chromatin interaction frequency by carrying out the Hi-C experiments in *Longissimus dorsi* muscle. Four individuals with two biological replicates of each stage generated 4.42 billion clean paired-end reads in total (Table S1). The Pearson’s correlation *r* of biological and technical replicates both ranged from 0.95 to 0.97 at 200 kb resolution (Fig. S1A), and we therefore merged the data for a higher-resolution analysis. A total of 1.22 billion unique mapped contacts passed all filters (Table S1), which were then used to construct raw and ICE [64] normalized matrices. The fetal cattle muscle (CFM) and adult cattle muscle (CAM) appeared to be similar to each other in genome-wide heatmap (Fig. 1A), which was supported by Pearson’s correlation of ICE matrices with *r* = 0.98 and *r* = 0.93 at 1 Mb and 40 kb, respectively (Fig. 1B and S1B).

We computed contact probability curves from 40-kb binned raw matrices. A rapid exponential decrease of contact frequencies was observed at short distances, especially for CAM (Slope, S_CAM_ = − 0.67, S_CFM_ = − 0.79) (Fig. S1C). Interestingly, an unexpected increase of contact probability at or after 10 Mb was detected, but CAM chromatin presented higher interaction frequency than CFM chromatin (Fig. S1C). The result suggested that CFM may have more short-range interactions, while CAM has more long-range interactions, which is similar to previous studies on 3D genome organization during mESC differentiation [65]. Additionally, we found a substantial number of different pairwise interactions by subtracting CAM ICE matrix from CFM ICE matrix at 1 Mb (Fig. S1D). Inter-chromosomal contact was also analyzed, given that the *trans-*interaction accounted for ~ 40% of total valid pairs (Table S1). We observed unequal distributions of inter-chromosomal contacts in both CFM and CAM genomes, such as strong interactions of chromosome 25 (Chr25) with Chr18 and Chr19, and significant differences in inter-chromosomal contacts between the two groups, such as CFM-specific strong interactions between Chr1 and Chr29 (Fig. S1E). To determine the resolution capability of our Hi-C data, we searched for the smallest bin size where 80% of bins have at least 1000 valid contact pairs [37]. We found that both CFM and CAM data could reach at least 10 kb resolution (Fig. 1C). The high quality and resolution of our Hi-C data enables us to explore the 3D genome dynamics during cattle muscle development at a fine scale.

### Chromatin dynamics with transcriptome changes

**Fig. 2:**
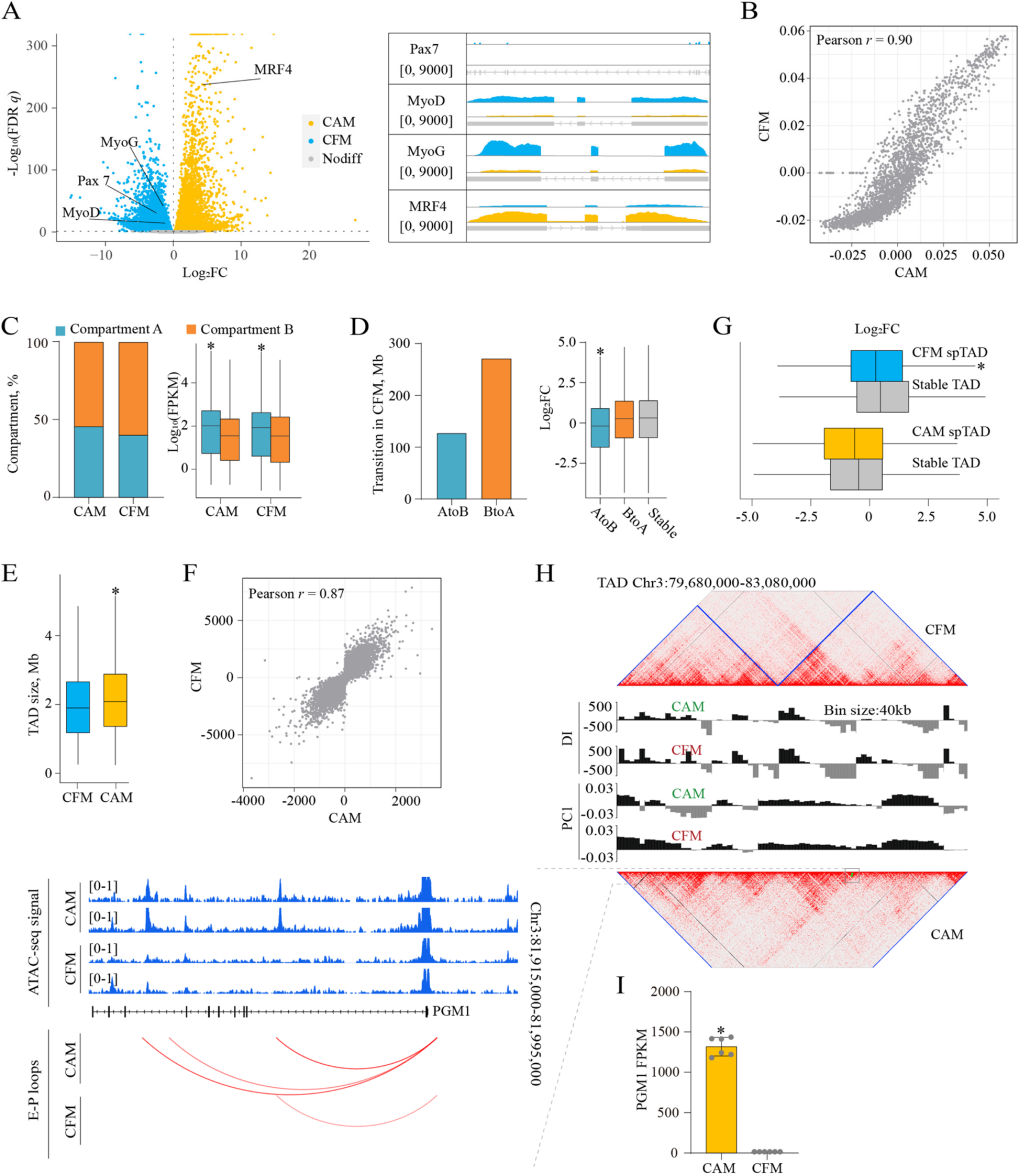
Re-organization of compartment and topologically associated domain (TAD) during cattle muscle development. **A** Volcano plot of differentially expressed genes (DEGs) with RNA-seq signal intensity for key myogenic regulators. **B** Pearson’s correlation of first principal component (PC1) values at 1 Mb resolution. **C** Statistics and transcriptional effects of A and B forms. **D** Statistics and transcriptional effects of compartment transition. **E** Boxplot of TAD size with average length of 2102.97 kb and 2224.51 kb in CFM and CAM, respectively. **F** Pearson’s correlation of directionality indices (DIs) at 40 kb. **G** The effects of split TADs (spTADs) on gene expressions. **H** and **I** An illustrated example of spTAD. CFM, fetal cattle muscle; CAM, adult cattle muscle. ^*^P < 0.05

Previous studies have showed that genome structural reprogramming is involved in embryonic development and myogenic differentiation in mouse [27, 66]. We first performed RNA-seq to associate gene expressions with chromatin dynamics. After data quality control (Fig. S2A, Table S2), we identified 13,933 DEGs (FDR *q-*value < 0.05), including 397 novel genes (Fig. 2A, Table S3). The expression profiles of key myogenic regulators were as expected (Fig. 2A). *Pax7* is responsible for lineage specification; MyoD commits cells to the myogenic program; *MyoG* initiates the terminal differentiation to myocytes with *MyoD* downregulation; *MRF4* functions in the formation of myotubes [67, 68].

To define the compartments and their transitions during development, we called the PC1 values of ICE matrices at 1 Mb resolution (Fig. S2B, Table S4). Overall, compartments showed high correlation between CFM and CAM (Pearson’s correlation *r* = 0.90) (Fig. 2B). A and B compartment forms accounted for 40.02% and 59.98% of fetal genome, respectively, while that were 45.50% and 54.50% in adult genome (Fig. 2C). Compared with B forms, A forms had a significantly higher gene expression level in both fetal and adult genomes (Wilcoxon’s test, *P*-value < 2.2e-16) (Fig. 2C). A total of 127 Mb regions in fetal genome were subject to transition from A to B form, accompanied with downregulation of 537 genes covered (Wilcoxon’s test, *P*-value = 1.28e-11), but B to A form transition of 271 Mb regions did not increase the expressions of 2717 genes (Wilcoxon’s test, *P*-value = 0.57) (Fig. 2D). Notably, the up-regulation of *MRF4* gene coordinated the elevated PC1 value in CAM, while the down-regulations of *Pax7*, *MyoD*, and *MyoG* were accompanied with the lowered PC1 value in CFM (Tables S3 and S4).

To define TAD structures, we calculated the DIs at 40 kb and identified 1130 and 1061 TADs in CFM and CAM, respectively (Fig. S2C, Table S5). The average length of CFM TADs was smaller than that of CAM TADs (2102.97 vs. 2224.51 kb, Wilcox’s test, *P*-value = 7.7e-03, Fig. 2E), which supported the findings in contact probability curves that CAM has more long-range interactions. We then directly compared the DIs of the two groups and found they were strongly correlated (Pearson’s correlation *r* = 0.87) (Fig. 2F). By defining the regions less than 400 kb in between TADs as topological boundaries, we identified only 38 different boundaries between the two groups (Table S6). These stage-specific TAD boundaries contained a total of 61 genes which were significantly enriched for embryonic morphogenesis, fatty acid metabolism, and regulation of protein dephosphorylation (*P*-value < 0.01), such as *SALL1*, *BOD1L1*, and *GJA1*.

Interestingly, we found some TADs were divided into two or more TADs between CFM and CAM, which was supported by previous findings during mammalian embryogenesis [66]. Here, we called the larger ones as unchanged TADs (ucTADs) and the smaller ones as split TADs (spTADs). There were 33 CFM spTADs and 19 CAM spTADs, corresponding to 16 and 9 ucTADs in CAM and CFM, respectively. Further analysis showed that the formation of fetal spTAD inhibited the expression of the inhabited genes (Wilcox’s test, *P*-value = 3.45e-02), and this trend was also observed in adult spTAD (Wilcox’s test, *P*-value = 0.39) (Fig. 2G). One possible reason is that active chromatin and transcription could affect the formation of 3D structures [69]. For example, *PGM1* gene in adult ucTAD had elevated levels of chromatin accessibility and transcription (Fig. 2H and I), compared to that in fetal spTAD, but communications across spTAD boundary between *PGM1* and regulatory elements were not observed in either ucTAD or spTAD (i.e. no hijacking mechanism [70].

These data demonstrated that the compartment and TAD structures undergo re-organization during cattle muscle development, which is significantly associated with transcriptome changes.

### Genome wide *cis*-regulatory elements and target genes annotation

**Fig. 3:**
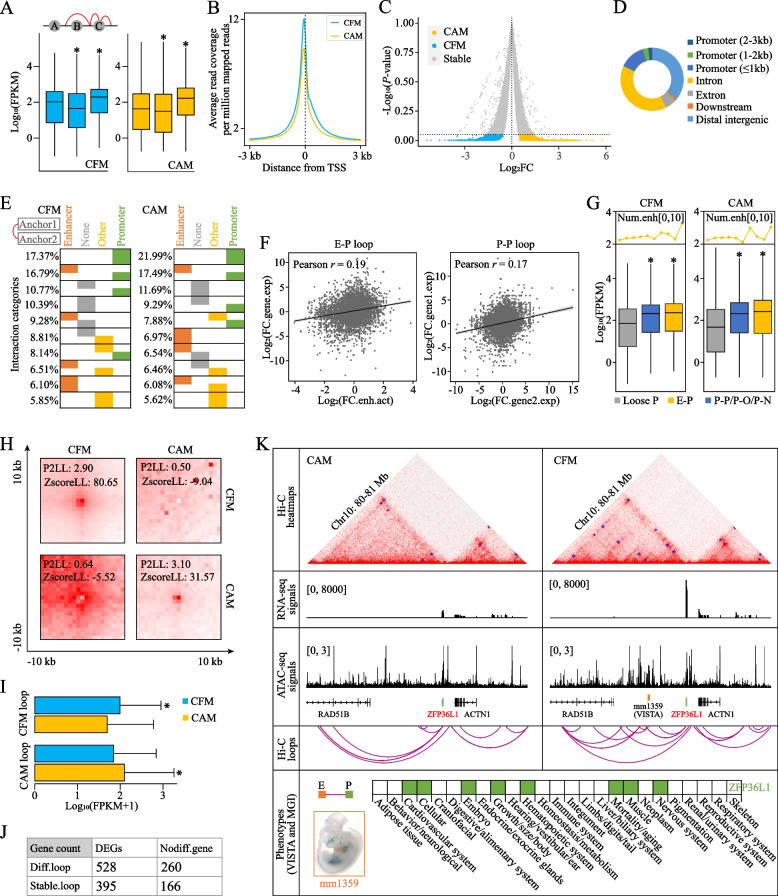
*Cis*-regulatory elements analysis. **A** Loop affected the expressions of genes of different types. **B** Average read coverage around transcription start sites (TSSs). **C** Volcano plot of differentially accessible regions (DARs). **D** Distribution of DAR. **E** P-P and E-P were the top two most frequent interaction categories during muscle development. **F** Pearson’s correlation between anchored elements. **G** E as well as P/O/N raised the expressions of target genes. Gene up-regulation was broadly accompanied by enhancer number increase. **H** Aggregate peak analysis (APA) of stage-specific loops. **I** and **J** The stage-specific loops elevated the expression levels of their anchored genes in both CFM and CAM. **K** An identified E-P loop putatively functions in cattle muscle development, supported by the evidence from VISTA (mm1359) and MGI (ZFP36L1). CFM, fetal cattle muscle; CAM, adult cattle muscle. ^*^P <0.05

Defining the target genes of *cis*-regulatory elements has been challenging as they frequently control distant rather than adjacent genes [71]. Chromatin loop could bring *cis*-regulatory elements to their cognate gene promoters within the same TAD, which acts as another layer of transcriptional regulation at a fine-scaled level (dozens to hundreds of kb) [17]. To characterize *ci*s-regulatory elements and their target genes, we first called intra-chromosomal loops using the HICCUPS program [34]. A total of 6322 and 9719 loops in CFM were identified at 5 and 10 kb, respectively, while 7154 and 8536 CAM loops were detected at the two resolutions, respectively. When merged, we got 10,575 fetal and 10,078 adult loops with significant difference in average length (322.75 vs. 278.56 kb, Wilcox’s test, *P*-value = 1.60e-10) (Table S7). To probe the transcriptional effects of loop structures, we categorized genes into three groups according to their promoter (defined as ±3 kb of transcription start sites) locations relative to loops: ‘A’ genes outside of loops, ‘B’ genes inside of loops, ‘C’ genes overlapped with loop anchors (Fig. 3A). Integrative analysis revealed that chromatin looping clearly increased ‘C’ gene expressions but decreased ‘B’ gene expression in both CFM and CAM when compared with that of ‘A’ genes (Wilcox’s test, *P-*value < 2.2e-16) (Fig. 3A).

Next, ATAC-seq was performed to annotate regulatory loops (i.e., interactions between regulatory elements). ATAC-seq could map chromatin accessibility landscape which reflects the primary positions of functional elements and is critical determinant of chromatin organization and function [72, 73]. After quality control (Fig. S3A and B, Table S8), we obtained 81,369 and 82,042 accessible regions in two CFM samples and 39,232 and 44,358 accessible regions in two CAM samples (Table S9). These peaks were enriched around the TSSs as expected (Fig. 3B). We then constructed a union peak set and identified 16,232 DARs (*P*-value < 0.05) between CFM and CAM (Fig. 3C and S3C, Table S10). These DARs were mainly mapped to introns (39.08%) and distal intergenic regions (35.94%) (Fig. 3D) and drastically enriched for motifs of key myogenic regulators (e.g., Pax7, MyoD, MyoG, and MRF4) (Fig. S3D, Table S11). The high-quality ATAC-seq data were subsequently used to compile the landscape of *ci*s-regulatory elements of cattle muscle genome. We integrated the 19,261 PSYCHIC 25-kb enhancers (Table S12) and 24,004 RNA-seq 6-kb promoters with the ATAC-seq union peaks and identified 14,980 enhancer peaks (E) and 16,391 promoter peaks (P), leaving 25,578 union peaks as other regulatory peaks (O) (Table S13). The 56,949 *ci*s-regulatory elements were wired to their targets by mapping them to 28,798 unique anchors of loop structures, resulting in extra annotation of 12,500 regulatory elements (i.e., anchors overlapped none of the ATAC-seq union peaks, N). We found at last 16,663 and 16,497 interaction pairs between *ci*s-regulatory elements in CFM and CAM, respectively. P-P (7.37% ~ 21.99%) and E-P (16.79% ~ 17.49%) were the top two most frequent interaction categories during muscle development, while the third types were N-P (10.77%) and N-N (11.69%) in CFM and CAM, respectively (Fig. 3E). Further analyses revealed positive correlation between enhancer activities and mRNA abundances (Pearson’s correlation *r* = 0.19, *P*-value < 2.2e-16), as well as co-expression of anchored genes (Pearson’s correlation *r* = 0.17, *P*-value < 2.2e-16), when *ci*s-regulatory elements were looped (Fig. 3F). In details, genes with E-P interactions had higher expression levels than loose genes (i.e., unlooped with regulatory elements) in both CFM and CAM (Wilcoxon’s test, *P*-value < 2.2e-16); genes involved in other loops (P-P, P-O, P-N) also had higher expression levels in both CFM and CAM (Wilcoxon’s test, *P*-value < 2.2e-16); but the transcriptional effects of E-P loops seemed higher than that of other loops (P-P, P-O, P-N) in both CFM (Wilcoxon’s test, *P*-value = 5.61e-2) and CAM (Wilcoxon’s test, *P*-value = 7.12e-2) (Fig. 3G). Besides, we found a broad tendency for gene expression to be raised as the number of looped enhancers increasing (Fig. 3G).

There were 517 and 888 stage-specific loops in CFM and CAM (Table S14), respectively. Aggregate peak analysis revealed peak enrichments (P2LL > 1 and ZscoreLL > 0, Fig. 3H), indicating good accuracy of our loop calling [37]. The stage-specific loops elevated the expression levels of their anchored genes in both CFM (Wilcox’s test, *P*-value = 6.47e-9) and CAM (Wilcox’s test, *P*-value = 1.06e-9) but were not enriched for DEGs (Fisher’s exact test, *P*-value = 0.19) (Fig. 3I and J), suggesting the complexity of gene regulations. There were 240 E-P interactions (Table S15) mediated by the stage-specific loops, where the anchored genes were strongly enriched for muscle development and the wired enhancers preferentially harbored motifs of MEF2, KLF, CTCF, MyoD, MyoG, and other key muscle transcription factors (Tables S16 and S17).

Here, we illustrated a delicate example of E-P interactions putatively involved in cattle muscle development (Fig. 3K). The RNA binding proteins ZFP36L1 functions in myogenesis [74] and embryonic growth (MGI database), etc. VISTA conserved element mm1359 within loop anchor was stained in branchial arch (8/9), heart (4/9), and abdominal region (5/9) at e11.5 (VISTA database). In summary, we established the landscape of *cis*-regulatory elements and revealed the effects of E-P loops on gene transcriptions, which provides key insights into cattle muscle biology.

### *Cis*-regulatory elements are enriched in selection sweeps

We performed selection sweep analysis using 86 cattle individuals from high beef production cattle and low beef production cattle breeds (Table S18). All the selected LBPC breeds were Chinese native breeds and their brief introductions can be retrieved from Catalogue of national livestock and Poultry Genetic resources (https://zypc.nahs.org.cn/pzml/).

**Fig. 4:**
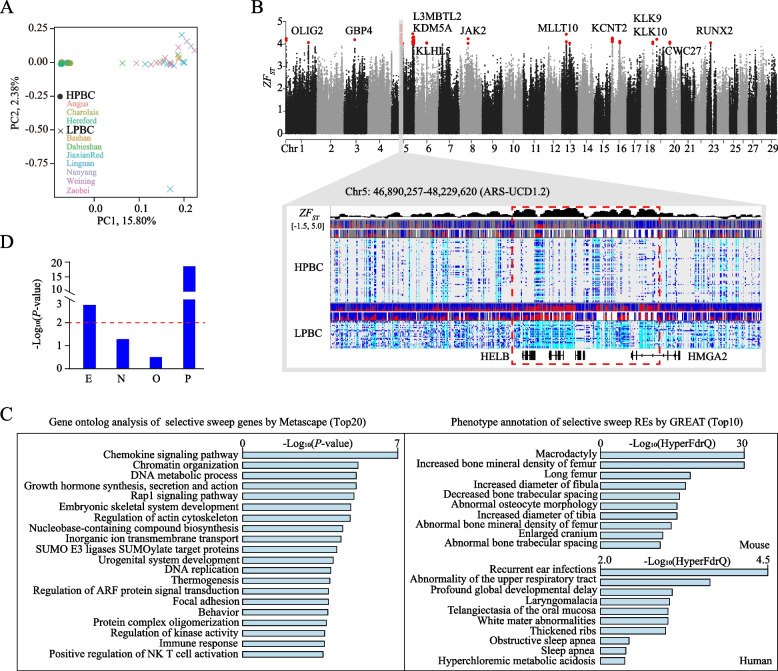
Selection sweep analysis. **A** Principal component analysis (PCA) of 60 high beef production cattle (HBPC) and 26 low beef production cattle (LBPC). **B** Manhattan plot of *ZF*_*ST*_ with strong signal on chr5: 47,490,158–47,883,745. **C** Functional annotations of genes and regulatory elements within the sweep regions. **D** Enrichment analysis of the sweep regions for *cis*-regulatory elements

After quality control and filtering, we got 26,908,820 SNPs. Principal component analysis clearly discriminated the two groups with PC1 and PC2 components as 15.8% and 2.38%, respectively (Fig. 4A). A total of 2045 sweep regions were identified (*ZF*_*ST*_ > 3, top ~ 0.4%) (Table S19). A strong selection signal was observed on chr5: 47,490,158–47,883,745 (ARS-UCD1.2) which covered *HELB*, *IRAK3*, *TMBIM4*, *LLPH*, and *HMGA2* (Fig. 4B). Notably, *HMGA2* gene has caused extreme events of evolution in horse, chicken, rabbit, dog and bird [75–80]. The *HMGA2* knockout mice and pigs present pygmy phenotype and deficiency in muscle growth [81–83]. Additionally, GWAS have identified *HMGA2* gene as promising candidate of cattle stature and beef production [84–87]. We also characterized the biological functions of all genes identified at selection signals (Table S20). These selection sweep genes were strongly enriched in gene sets related to chemokine signaling pathway, chromatin organization, growth hormone synthesis, secretion and action, and embryonic skeletal system development (Fig. 4C).

LOLA [61] was used to evaluate the regulatory elements against the distribution of sweep regions. We found strong co-localization of the two genomic region sets (Fig. 4D). Two components of regulatory elements were significant: promoters (Fisher’s exact test, *P*-value = 1.74e-19) and enhancers (Fisher’s exact test, *P*-value = 1.60e-3), supported by previous findings that regulatory elements contributed to the domestication of sheep and cotton [88, 89]. There were 616 regulatory elements within sweep regions, including 178 promoters (P), 138 enhancers (E), 189 other ATAC-seq peaks (O), and 111 anchors without any ATAC-seq peaks (N) (Table S20). These regulatory elements were enriched in mouse bone growth and human global development, interpreted by GREAT [60] with all the identified regulatory elements as input background (Fig. 4D). These data suggested that *cis*-regulatory elements within selection signals probably contributed to the meat production divergence between Chinese native and international reputed beef cattle breeds apart from coding gene candidates.

### HMGA2 intronic enhancer affects PBM proliferation

**Fig. 5:**
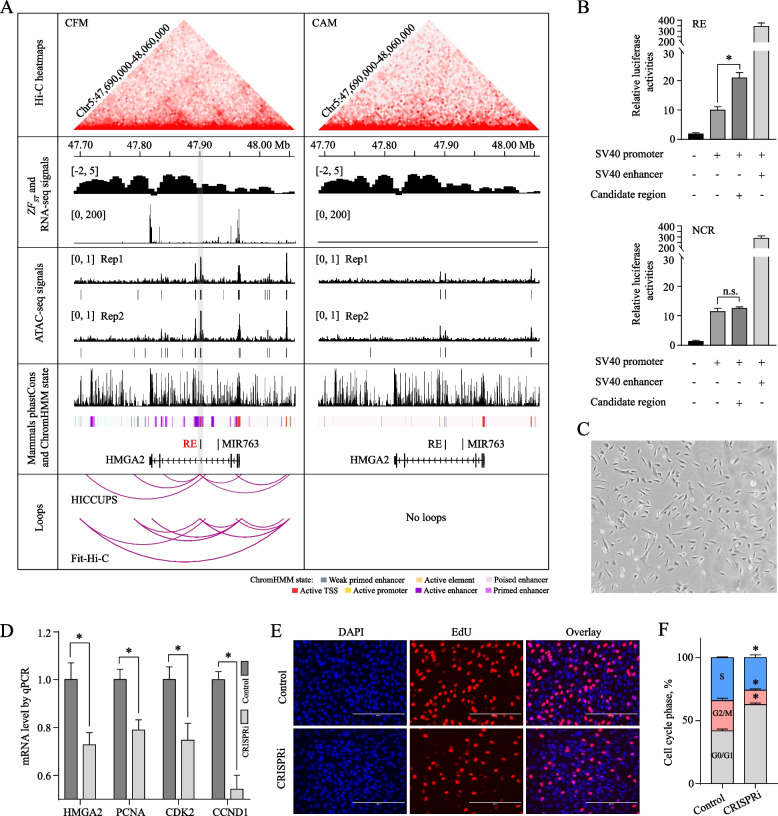
The regulatory effect of RE on primary bovine myoblast (PBM) proliferation. **A** Visualization of integrative information about RE (highlighted in gray bar). **B** Validation of RE enhancer activity using a Dual-Luciferase Reporter System in PBMs. Data shown as means ± SD (*n* = 5). **C** PBMs isolated from *Longissimus dorsi* of fetal cattle. **D**–**F** Validation of the effects of RE on PBM proliferation using a CRISPR-mediated interference (CRISPRi) system. The expressions of *HMGA2* and proliferation markers were significantly decreased (*n* = 9) (**D**). The number of EdU positive cells was clearly reduced (**E**). The percentage of PBMs in S phase was considerably decreased (*n* = 3) (**F**). CFM, fetal cattle muscle; CAM, adult cattle muscle; NCR, negative control region. ^*^P < 0.05

The *HMGA2* gene, coding an architectural transcription factor, functions conservatively in organism development across various species. It plays crucial roles in but not limited to the self-renew of diverse stem cells, such as muscle satellite cell, haematopoietic stem cells, and myeloerythroid progenitor [82, 90–92]. Several lines of evidence, taken together, show that this gene is involved in LIN28-let-7-HMGA2-IGF2BP2 axis and HMGA2-PLAG1-IGF2 pathway, which endows HMGA2 with pleiotropic effects [93–95]. Although the mechanisms of action of HMGA2 have been well established, studies on *HMGA2* regulatory elements are still limited [96]. In this study, 6 regulatory elements were identified around/in *HMGA2* and one of them could interact with *HMGA2* promoter, predicted by both HICCUPS [34] (5 kb and 10 kb resolution) and Fit-Hi-C [39] (10 kb resolution) (Fig. 5A and S4, Tables S7 and S21). This regulatory element (chr5: 47,901,023–47,902,297) was annotated as putative enhancer in our data and had frequent interactions with other functional regions (Fig. 5A and S4).

Ruminant Genome Database (http://animal.nwsuaf.edu.cn/code/index.php/RGD) deposited this regulatory element as poised and active enhancer in adult skeletal muscle and fetal rumen epithelial primary cells, respectively (Fig. 5A). We named this putative enhancer as RE and performed experimental validation.

First, Dual-Luciferase Reporter System was used to test the enhancer activity of RE. Adding RE to pGL3-Promoter could significantly trigger relative luciferase activity in PBMs (two-sided Student’s *t*-test, *P*-value = 3.00e-6), while adding a negative control region (chr5: 47,936,013–47,936,580) without any ATAC-seq peaks did not work (two-sided Student’s *t*-test, *P*-value = 0.09) (Fig. 5B and C, Table S22). This result confirmed the enhancer activity of RE in PBMs. Next, we applied the CRISPRi system, which fused inactive Cas9 to the KRAB transcriptional repressor domain to repress regulatory elements [97], to explore the effects of RE on PBM proliferation. RE repression resulted in down-regulation of proliferation markers, *PCNA* (two-sided Student’s *t*-test, *P*-value = 3.87e-3), *CDK2* (two-sided Student’s *t*-test, *P*-value = 7.73e-3), and *CCND1* (two-sided Student’s *t*-test, *P*-value = 2.93e-4), accompanied by a modest yet significant reduction (~ 25%) in *HMGA2* expression (two-sided Student’s *t*-test, *P*-value = 5.26e-3) (Fig. 5D, Table S22). EdU proliferation assay indicated that the number of EdU positive (S-phase, red) cells clearly decreased after RE CRISPRi (Fig. 5E), which was supported by the result of flow cytometry that proliferative PBM amount was reduced by ~ 10% (one-way ANOVA with Dunnett multiple comparisons test, *P*-value = 3.63e-3) (Fig. 5F and S5, Table S22). These results demonstrated that RE could regulate *HMGA2* expression and PBM proliferation.

## Discussion

Chromosome territory hypothesis was proposed about one hundred years ago and validated with fluorescence in situ hybridization after about eight decades later, thereby instigating the study of nuclear architecture [98]. The advent of high-throughput sequencing of chromosome conformation capture propelled the study of nuclear architecture at high resolution and, therefore, into the era of 3D genomics [11, 99]. Today, 3D genome study is committed to unravel the regulatory mechanism of chromosome organization and gene expression, as well as how this is coupled to 3D nuclear organization [100].

Using long-range interaction data, 3D genomic data benefits de novo reference assembly, whole-genome haplotype reconstruction, and the identification of target genes of GWAS hits in non-coding region, other than chromatin structure annotation, which holds high promise to understand the genetic basis of economic traits of farmed animals [101–103]. While the studies of 3D genome have been reported for livestock species across diverse tissues and cell lines, information about cattle muscle has not been reported yet [21, 22, 30, 104–108].

In present study, we performed Hi-C with in-depth sequencing (around 5 ~ 10 kb resolution) to identify the hierarchic chromatin structures, including compartment, TAD, and loop. To the best of our knowledge, it is the first study about the comparative genome conformations of muscle tissues from fetal and adult cattle, which provides a foundational dataset for the functional characterization of cattle genome.

Our results demonstrated the dynamics of chromatin structures during muscle development. At the compartment level, we observed moderate compartment reorganizations which was comprised only ~ 14.6% of genomic regions. During the differentiation of myoblasts and iPax7 progenitors, the scales of compartment switches account for 8% and ~ 6.5% of mouse genome, respectively [27, 29]. At the TAD level, we obtained ~ 1000 TADs in both CFM and CAM, but only a few TAD boundaries were changed, which agreed with the findings during iPax7 progenitor differentiation and chicken folliculogenesis [22, 27]. This confirmed that TAD is a genuine unit of chromatin packing [109]. At loop level, we identified ~ 10,000 loops in both CFM and CAM with 1405 of them as stage-specific loops. There were 240 E-P interactions involved in these stage-specific loops. These anchored genes were strongly enriched for muscle development, such as *ACTN2*, *SMAD7*, *TMOD1*, and *ZBTB18* [110–113], while the looped enhancers were drastically enriched for motifs of CTCF and key myogenic regulators, such as MEF2, KLF, MyoD, and MyoG.

We then integrated Hi-C interaction data maps with transcriptome and found two interesting results. The formation of spTAD seemed to inhibit gene expressions. It was reported that *HPV-CCDC106* integration splits a local TAD into two smaller TADs, accompanied by an enhancer hijacking event to form a novel loop structure to increase *CCDC106* expression in cervical cancer [70]. The enhancer hijacking event was not observed at *PGM1* locus, but instead the chromatin accessibility was significant changed. Chromatin state and transcription may contribute to the spTAD formation [69, 114–116]. Another interesting finding is that looping could up-regulate the expressions of anchor genes, especially genes looped with enhancers, but unexpectedly down-regulate the expressions of genes within loops. The suppression effects may be required for precise regulation of gene expression by avoiding promoter competition [117, 118]. This observation together with the formation of spTAD raises an interesting question that which is the initial cause for genome function and architecture. More recently, it has been proposed that genome function is a major driver of genome architecture and that structure facilitates, but does not determine, function [119]. Indeed, loss of cohesion, a key protein lying at loop anchors and TAD boundaries and regulating genome folding, eliminates loop domains but does not lead to widespread changes in gene expressions [120]. Emerging evidences indicate that sequence features, histone modification, transcription factor binding, and phase separation, other than transcription, also affect chromosome organization [121–127]. These facts suggest the complex mechanoregulation of gene expression coupled to the dynamics of 3D genome. Hence, it is not surprising that the stage-specific loops were not enriched for DEGs compared with stable loops.

In this study, we annotated regulatory elements for cattle muscle at two developmental stages, using ATAC-seq, RNA-seq, and Hi-C. Although several studies have established the regulatory element profiles for cattle across diverse tissues, including liver, rumen, oocyte and early embryo, data about cattle muscle tissue is still limited [128–130]. Stage- and tissue-specific information is required to better understand the genetic basis of phenotypes. More recently, 6 epigenetic data types have been profiled in 8 tissues for adult male cattle, but the fetal cattle datasets were absent [131]. In this study, we identified 56,949 *ci*s-regulatory elements in CFM and CAM, which is comparable to that previous study (on average ~ 150,000 regulatory elements in eight tissues). Interestingly, enhancer and promoter were significantly enriched in selection sweeps which were identified by comparing high with low beef production cattle breeds. It has been reported that enhancer evolution is a universal feature of mammalian genomes and can be associated with genes under positive selection [3, 128].

Finally, we performed experimental validation of a putative enhancer within *HMGA2* gene which overlapped with a strong selection signal. Dual-Luciferase reporter assay confirmed the enhancer activity of RE in PBMs. The assays of qPCR, EdU, and flow cytometry with CRISPRi system revealed the positive effects of RE on *HMGA2* expression and PBM proliferation. Notably, in addition to *HMGA2* promoter, other functional regions located at the strong sweep region could interact with RE, suggesting the complicated mechanism underlying *HMGA2* transcription. Indeed, many super-enhancers have been mapped to *HMGA2* locus of human and mouse across diverse tissues and cell lines (SEA Version 3.0: Super-Enhancer Archive [132]). The functional relevance of other regulatory elements at *HMGA2* locus in regulating *HMGA2* and RE needs further investigation.

## Conclusion

In this study, we constructed the first dynamic map of genome conformations of muscle tissues from fetal and adult cattle. We found a general pattern of chromatin organization accompanied by transcriptomic change during cattle muscle development. The enhancers and promoters, annotated by interaction data, were enriched in selection sweeps, suggesting that *cis*-regulatory elements probably contributed to the meat production divergence between Chinese native and international reputed beef cattle breeds. Our results provide a foundational dataset for the functional characterization of cattle genome.

## Supplementary Information

**Additional file 1: Table S1.** Hi-C library stats of CFM and CAM. **Table S2.** RNA-seq library stats of CFM and CAM. **Table S3.** Gene expression levels in CFM and CAM. **Table S4.** The first principal component (PC1) values of CFM and CAM at 1 Mb resolution. **Table S5.** The locations of topologically associated domains (TADs) of CFM and CAM at 40 kb resolution. **Table S6.** Stage-specific TAD boundaries of CFM and CAM at 40 kb resolution. **Table S7.** Loop list of CFM and CAM after merging loops at 5 kb and 10 kb resolutions. **Table S8.** ATAC-seq library stats of CFM and CAM. **Table S9.** Individual peak sets of four ATAC-seq samples. **Table S10.** Differentially accessible regions (DARs) between CFM and CAM. **Table S11.** Motif enrichment analysis of the differentially accessible regions (DARs). **Table S12.** Potential enhancers predicted by PSYCHIC at 25 kb resolution (FDR *q*-value < 0.05). **Table S13.** Landscape of *cis*-regulatory elements of cattle muscle genome based on ATAC-seq. **Table S14.** Stage-specific loops of cattle muscle. **Table S15.** E-P interactions involved in stage-specific loops. **Table S16.** Gene Ontology (GO) analysis of E-P genes in stage-specific loops using Metascape. **Table S17.** Motif enrichment analysis of E-P enhancers in stage-specific loops. **Table S18.** SRA ID of cattle used for selection sweep analysis. **Table S19.** Selection sweep signals (regions with *ZF*_*ST*_ > 3 were used for further analyses). **Table S20.** Genes and regulatory elements within sweep regions (*ZF*_*ST*_ > 3). **Table S21.** Fit-Hi-C loops (loops with FDR *q*-value < 0.05 were used for Fig. 5A). **Table S22.** Raw data of Dual-Luciferase reporter assay and PBM proliferation assay. **Table S23.** Primer sequences used in this study.

**Additional file 2: Fig. S1.** Basic characterization of 3D genome of fetal and adult cattle Longissimus dorsi muscle, related to Fig. 1. **Fig. S2.** Comparative maps of compartment and topologically associated domain (TAD), related to Fig. 2. **Fig. S3.** Data quality control and differentially accessible region (DAR) analysis for ATAC-seq. **Fig. S4.** Zoom-out features about RE for a wide view, related to Fig. 5A. **Fig. S5.** PBM proliferation assay (flow cytometry) using CRISPRi.

## Data Availability

All data are available within the manuscript and the electronic supplementary material. Raw sequencing data of Hi-C, ATAC-seq, and RNA-seq have been deposited in the NCBI Sequence Read Archive (SRA) under the Bioproject accession number PRJNA635966, PRJNA555664, PRJNA577592, respectively.

## Abbreviations

ATAC-seq: Assay for transposase accessible chromatin with high-throughput sequencing
CAM: Adult cattle muscle
CFM: Fetal cattle muscle
CRISPRi: CRISPR interference
DAR: Differentially accessible region
DEG: Differentially expressed gene
DI: Directionality index
EdU: 5-Ethynyl-2′-deoxyuridine
FDR: False discovery rate
FPKM: Fragments per kilobase of transcript per million fragments mapped
GWAS: Genome-wide association study
Hi-C: High-throughput chromosome conformation capture
HMGA2: High mobility group AT-hook 2
ICE: Iterative correction and eigenvector decomposition
PBM: Primary bovine myoblast
PCA: Principal component analysis
QC: Qinchuan cattle
RE: Enhancer candidate
RNA-seq: RNA sequencing
RT: Room temperature
SNP: Single nucleotide polymorphisms
spTAD: Split TAD
TAD: Topologically associating domain
TSS: Transcription start site
ucTAD: Unchanged TAD
*ZF*_*ST*_: Z-transformed fixation index
