# Supplementary material for: Dynamic chromatin architectures provide insights into the genetics of cattle myogenesis
Source: J Anim Sci Biotechnol. 2023 Apr 14;14:59. doi: 10.1186/s40104-023-00855-y (PMC10103417; doi:10.1186/s40104-023-00855-y)
Supplement: Supplementary file 2 — Additional file 2: Fig. S1. Basic characterization of 3D genome of fetal and adult cattle Longissimus dorsi muscle, related to Fig. 1. Fig. S2. Comparative maps of compartment and topologically associated domain (TAD), related to Fig. 2. Fig. S3. Data quality control and differentially accessible region (DAR) analysis for ATAC-seq. Fig. S4. Zoom-out features about RE for a wide view, related to Fig. 5A. Fig. S5. PBM proliferation assay (flow cytometry) using CRISPRi. [file 40104_2023_855_MOESM2_ESM.docx]

**
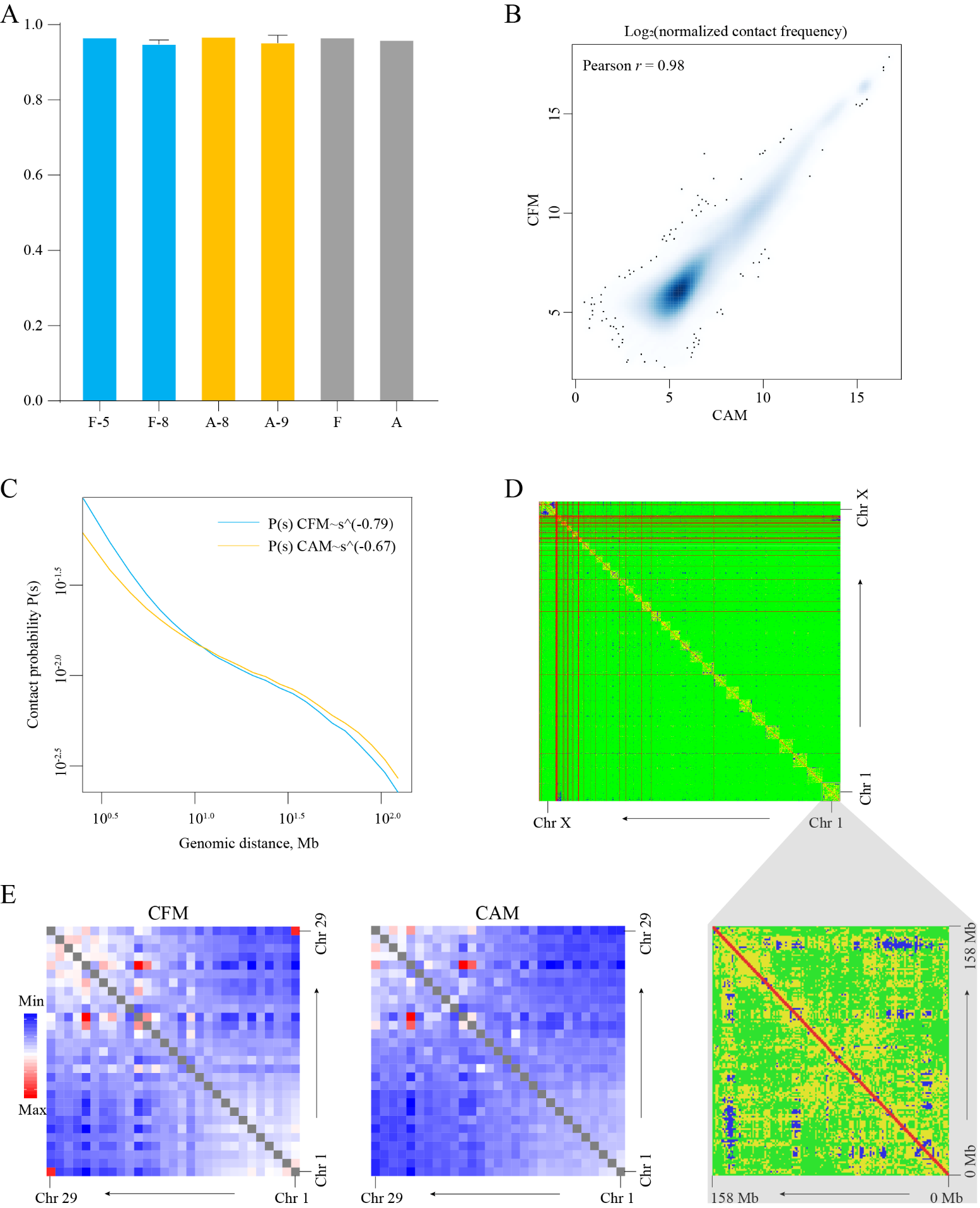
**

**Fig. S1** Basic characterization of 3D genome of fetal and adult cattle *Longissimus dorsi* muscle, related to Fig. 1. **A** Pearson’s correlation of biological and technical replicates at 200 kb resolution. **B** Pearson’s correlation between CFM and CAM ICE matrices at 1 Mb. **C** Plots of contact probability versus genomic distance with 40-kb binned raw matrices. **D** Heatmap of subtractive matrix at 1 Mb (CFM - CAM); blue: significant difference with FDR *q*-value < 0.05 and fold change ≥ 2 (92,745 pairwise interactions); yellow: significant difference with FDR *q*-value < 0.05 and fold change < 2 (160,815 pairwise interactions); green: no significant difference; red: regions excluded for comparisons. **E** Heatmaps of inter-chromosomal contacts showed by the value of observed/expected


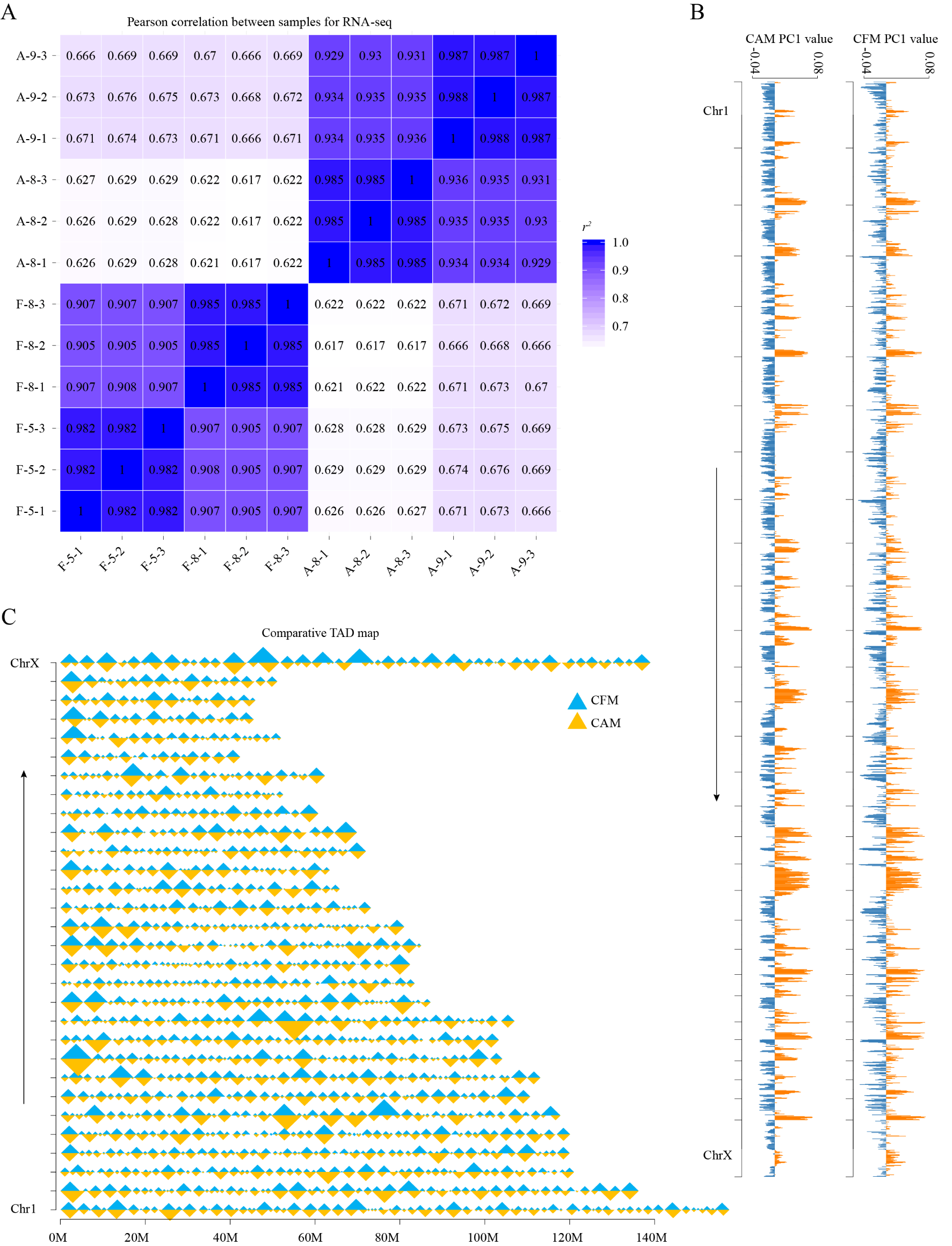


**Fig. S2** Comparative maps of compartment and topologically associated domain (TAD), related to Fig. 2. **A** Pearson’s correlation between samples for RNA-seq. **B** Visualization of the first principal component (PC1) values of CFM and CAM at 1 Mb resolution. **C** Visualization of the TADs of CFM and CAM at 40 kb resolution


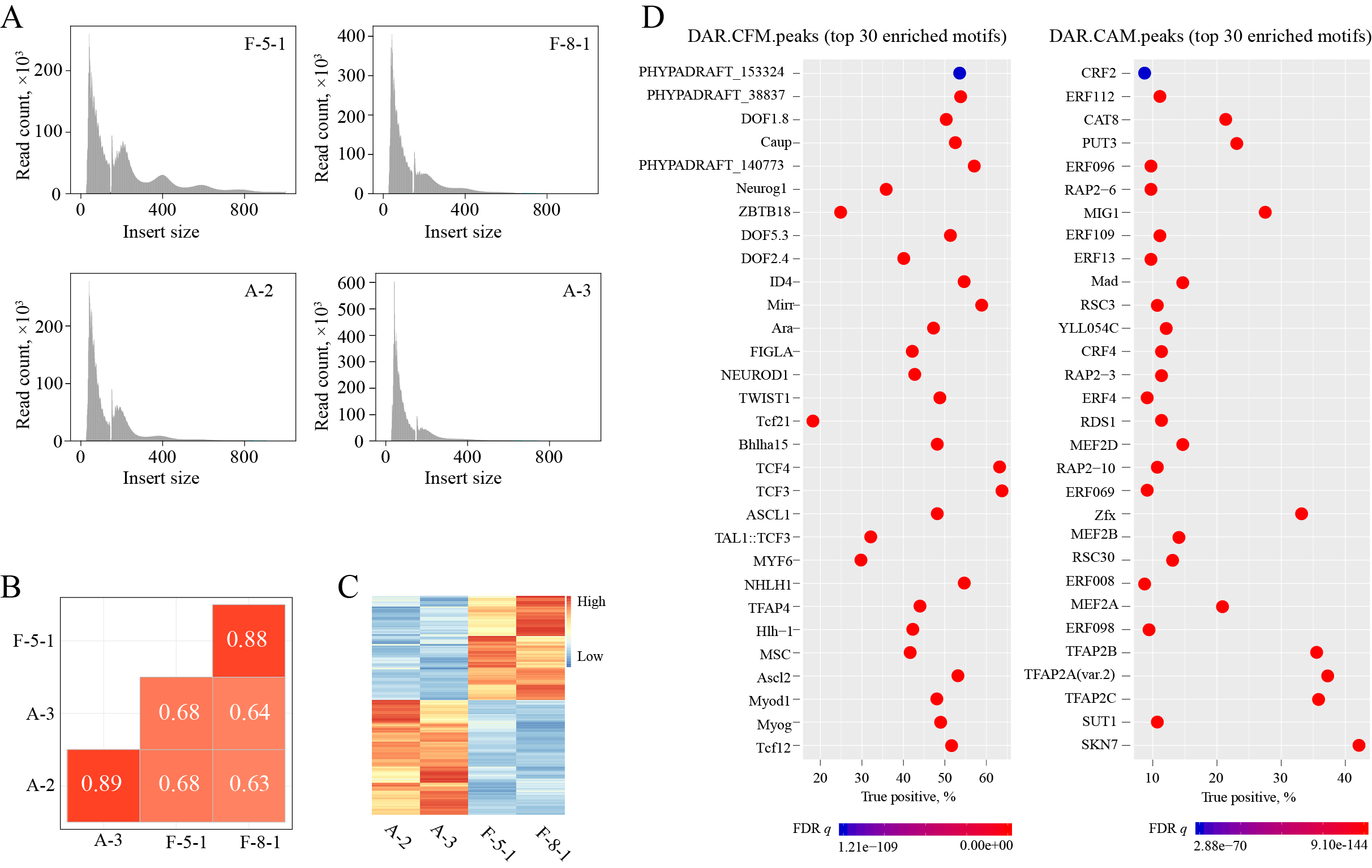


**Fig. S3** Data quality control and differentially accessible region (DAR) analysis for ATAC-seq. **A** The distribution of insert size had a periodicity of ∼200 bp. **B** Pearson’s correlation between samples for ATAC-seq. **C** Heatmap of 16,232 DARs (*P*-value < 0.05). **D** Top 30 enriched motifs of DARs (left: CFM peaks, right: CAM peaks)


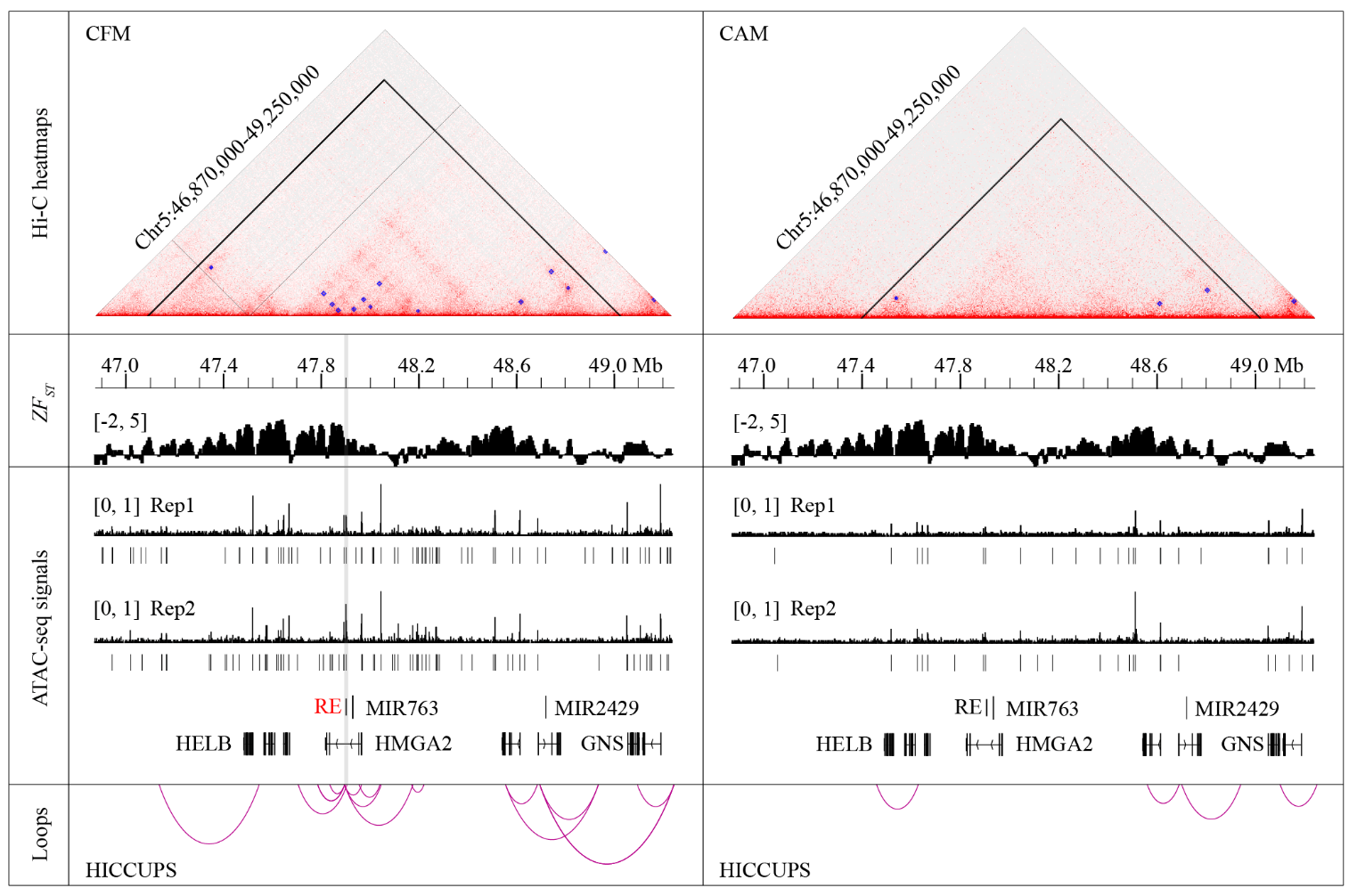


**Fig. S4** Zoom-out features about RE for a wide view, related to Fig. 5A. Topologically associated domains (TADs) shown as black triangular lines


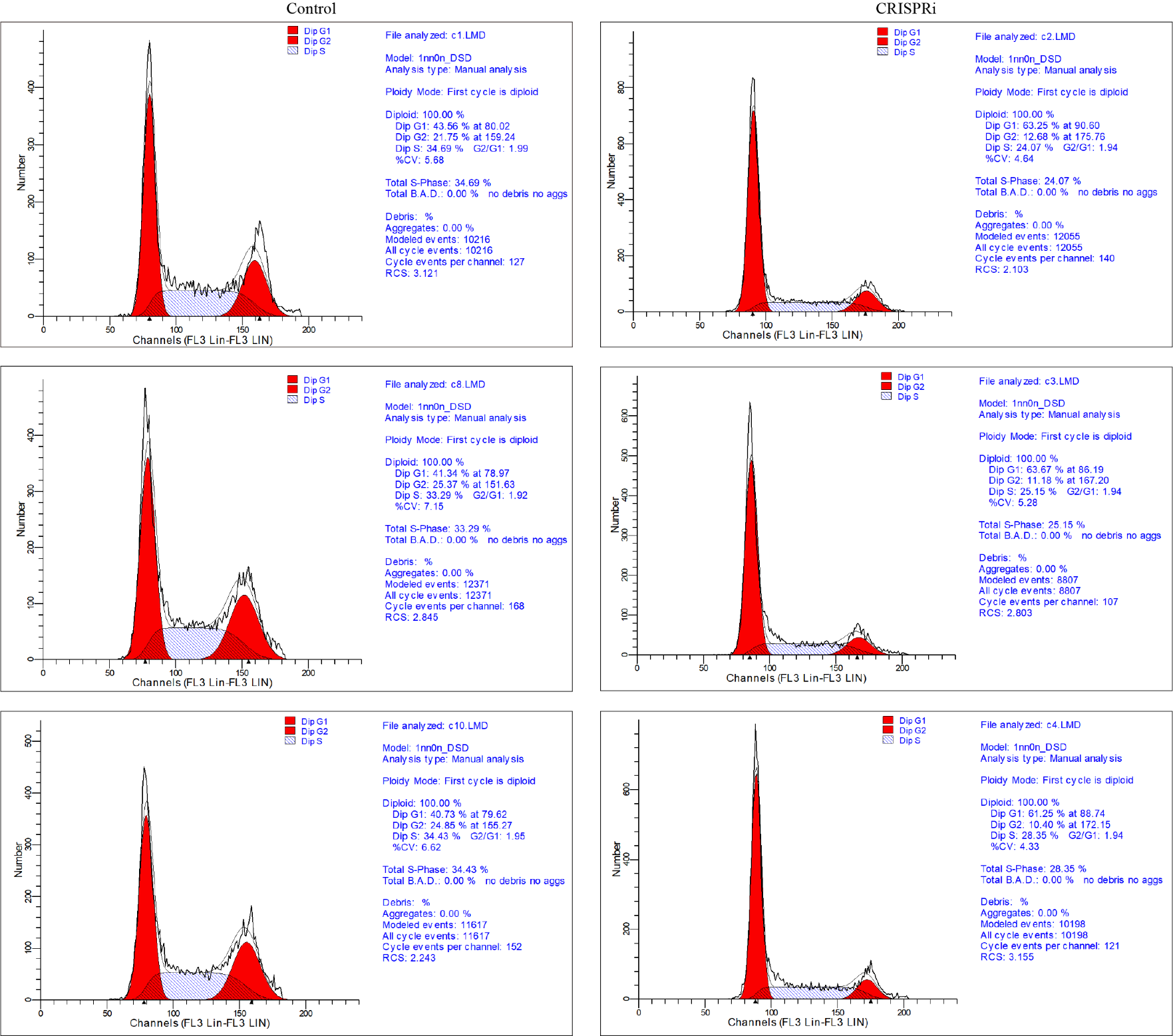


**Fig. S5** PBM proliferation assay (flow cytometry) using CRISPRi
